# Supplementary material for: Immune-Related Pneumonitis Was Decreased by Addition of Chemotherapy with PD-1/L1 Inhibitors: Systematic Review and Network Meta-Analysis of Randomized Controlled Trials (RCTs)
Source: Curr Oncol. 2022 Jan 9;29(1):267–82. doi: 10.3390/curroncol29010025 (PMC8774972; doi:10.3390/curroncol29010025)
Supplement: Supplementary file 1 [file curroncol-29-00025-s001.zip › curroncol-1464007-supplementary.pdf]

Supplementary Materials: Immune-Related Pneumonitis Was Decreased by Addition of Chemotherapy with PD-1/L1 Inhibitors: Systematic Review and Network Meta-Analysis of Randomized Controlled Trials (RCTs)

Table S1. Demographic Characteristics of the Patients at Baseline.

Characteristic	Total (n=11466)	I+C group (n=5406)	I group (n=6060)	*p*-value
**No. of RCT**	31 trails	15 trails	19 trails	0.424
**Median age**	64 years	63 years	65 years	N. R
**Sex**				
Male	6443(56.2%)	2384(44.1%)	4059(67.0%)	0.152
Female	5023(43.8%)	3022(55.1%)	2001(33.0%)	0.122
**History**				
NSCLC/SCLC	5616(49.0%)	2467(45.6%)	3149(52.0%)	0.748
TNBC	2269(19.8%)	1960(36.3%)	309(5.1%)	0.573
ES, G/GEJ	1112(9.7%)	250(4.6%)	862(14.2%)	0.401
UC	1419(12.4%)	453(8.4%)	966(15.9%)	0.141
Others	1050(9.1%)	276(5.1%)	774(12.8%)	0.775
**PD-1/L1 Inhibitors**				
PD-1 inhibitors	7481(65.2%)	2775(51.3%)	4706(77.7%)	0.263
PD-L1 inhibitors	3985(34.8%)	2631(48.7%)	1354(22.3%)	0.840

Abbreviation: I+C group, PD-1/L1 inhibitors plus chemotherapy; I group, PD-1/L1 inhibitors; NSCLC: non-small cell lung cancer; SCLC: small cell lung cancer; G/GEJ: Gastric and gastroesophageal junction cancer; TNBC: Triple-negative breast cancer; NSCLC: non–small-cell lung cancer; UC: urothelial cancer; HNSCC: Head and neck squamous cell carcinoma; ES: esophagus.

Table S2. Value of SUCRA and mean rank in overall population. Higher SUCRA scores correlated with a higher risk of IRP.

Treatment group	Any grade IRP		Grade 3-5 IRP	
	SUCRA	Mean rank	SUCRA	Mean rank
PD-1/L1+chemotherapy	50.7	2	50.5	2
PD-1/L1 monotherapy	99.3	1	99.2	1
Chemotherapy	50.7	3	0.3	3

Notes: IRP, Immune-related Pneumonitis; ICIs, immune check inhibitors; SUCRA, surface

under the cumulative ranking curve.

Table S3. Value of SUCRA and mean rank for different ICIs comparison. Higher SUCRA scores correlated with a higher risk of IRP.

Treatment group	Any grade IRP		Grade 3-5 IRP	
	SUCRA	Mean rank	SUCRA	Mean rank
PD-1 monotherapy	97.8	1.1	92.5	1.3
PD-1+chemotherapy	52.4	2.9	65.7	2.9
PD-L1 monotherapy	52.4	2.9	52.9	2.4
PD-L1+chemotherapy	47.3	3.1	34.0	3.6
Chemotherapy	0.0	5.0	4.8	4.8

Notes: IRP, Immune-related Pneumonitis; ICIs, immune check inhibitors; SUCRA, surface under the cumulative ranking curve.

Table S4. The value of SCURA for 3 group and mixed RR that I+C group Versus I group based on cancer type

Immune-related Pneumonitis	Any grade IRP				Grade 3-5 IRP			
	I+C group	I group	C group	RR (I+C VS. I)	I+C group	I group	C group	RR (I+C VS. I)
**Cancer type**								
NSCLC	51.3	98.7	0.0	0.44 (0.19-1.01)	49.7	98.9	1.4	0.39 (0.15-0.98)
TNBC	58.5	82.7	8.8	0.25 (0.00-14.09)	63.1	78.8	8.2	0.46 (0.02-12.13)
UC	45.1	97.4	7.5	0.23 (0.04-1.33)	N. R	N. R	N. R	N. R
G/GEJ/ES	70.6	79.1	0.1	0.91 (0.36-2.27)	N. R	N. R	N. R	N. R

Notes: I+C group, PD-1/L1 inhibitors plus chemotherapy; I group, PD-1/L1 inhibitors; NSCLC: non-small cell lung cancer; SCLC: small cell lung cancer; G/GEJ: Gastric and gastroesophageal junction cancer; TNBC: Triple-negative breast cancer; NSCLC: non–small-cell lung cancer; UC: urothelial cancer; HNSCC: Head and neck squamous cell carcinoma; ES, G/GEJ: esophageal cancer and gastric or gastroesophageal cancer.

Table S5. The value of P for inconsistency model test and IF for loop inconsistency in overall population and subgroup analysis.

Outcomes of Interest	Any grade IRP				Grade 3-5 IRP			
	No. of RCTs	*p*-value	Loop	95%CI IF	No. of RCTs	*p*-value	Loop	95%CI IF
**Overall population**	31	0.3931	0.684	(0.00-1.42)	27	0.9930	0.008	(0.00-1.84)
**ICIs**	31	0.3545	0.424	(0.00-1.87)	27	0.9616	0.047	(0.00-1.98)
**NSCLC**	17	N. R	N. R	N. R	16	N. R	N. R	N. R
**TNBC**	5	N. R	N. R	N. R	5	N. R	N. R	N. R
**UC**	3	0.1725	1.224	(0.00-3.15)	2	N. R	N. R	N. R
**ES, GC/GEJ**	3	0.9805	0.030	(0.00-2.65)	2	N. R	N. R	N. R

Notes: No.: number; NSCLC: non-small cell lung cancer; SCLC: small cell lung cancer; G/GEJ: Gastric and gastroesophageal junction cancer; TNBC: Triple-negative breast cancer; NSCLC: non–small-cell lung cancer; UC: urothelial cancer; HNSCC: Head and neck squamous cell carcinoma; ES, G/GEJ: esophageal cancer and gastric or gastroesophageal cancer; N.R, not report.

Table S6. Results of node-splitting analysis for the assessment inconsistencies for the IRP in cancer type.

Comparator	Intervention	Overall	NSCLC	TNBC	UC	G, G/GEJ
**Any grade IRP**						
C group	I group	0.26	N. R	N. R	N. R	N. R
C group	I+C group	0.11	N. R	N. R	0.17	0.98
I group	I+C group	0.12	N. R	N. R	0.17	0.98
**Grade 3 -5 IRP**						
C group	I group	0.99	N. R	N. R	N. R	N. R
C group	I+C group	0.99	N. R	N. R	N. R	N. R
I group	I+C group	0.99	N. R	N. R	N. R	N. R

Notes: IRP, immune-related pneumonitis; C group, chemotherapy group; I group, PD-1/L1 monotherapy; I+C group, PD-1/L1+chemotherapy. N. R, not report.

Table S7. **Results of node-splitting analysis for the assessment inconsistencies for the IRP in different ICIs group.**

Comparator	Intervention	***p*-value of any grade**	***p*-value of grade 3-5**
**IRP in different ICIs**			
Chemotherapy	PD-1 monotherapy	0.043	0.96
Chemotherapy	PD-1+chemotherapy	0.064	0.96
Chemotherapy	PD-L1 monotherapy	0.447	N. R
Chemotherapy	PD-L1+chemotherapy	0.970	N. R
PD-1 monotherapy	PD-1+chemotherapy	0.025	0.96
PD-L1 monotherapy	PD-L1+chemotherapy	0.644	N. R

Notes: IRP, immune-related pneumonitis; ICIs, immune check inhibitors; N. R, not report.

Table S8：Supplementary basic characteristic for eligible RCTs.

Study name	Study ID	Total, No.		Grade 1-5 IRP, No.		Grade 3-5 IRP, No.	
		Exp	Con	Exp	Con	Exp	Con
CASPIAN	NCT03043872	265	266	7	2	2	1
KEYNOTE-062	NCT02494583	250	254	8	9	N. R	N. R
IMpassion130	NCT02425891	453	437	16	1	2	0
KEYNOTE-522	NCT03036488	781	389	10	5	3	1
KEYNOTE-024	NCT02142738	154	150	12	1	4	1
KEYNOTE-604	NCT03066778	223	223	9	5	3	0
MYSTIC	NCT02453282	369	352	8	5	5	2
KEYNOTE-407	NCT02775435	278	280	23	6	9	3
KEYNOTE-045	NCT02256436	267	245	11	1	6	0
IMpassion031	NCT03197935	164	167	2	2	1	0
IMpower133	NCT02763579	198	196	5	5	N. R	N. R
IMpower110	NCT02409342	286	263	11	1	2	0
KEYNOTE-010	NCT01905657	682	309	40	6	18	2
KEYNOTE-189	NCT02578680	405	202	20	6	12	4
IMvigor130	NCT02807636	453	354	12	12	N. R	N. R
IMpower130	NCT02367781	473	232	31	3	0	1
KEYNOTE-042	NCT02220894	636	615	53	3	22	1
KEYNOTE-061	NCT02370498	294	276	8	0	2	0
KEYNOTE-048	NCT02358031	276	300	15	19	5	2
IMpower132	NCT02657434	291	274	18	6	6	3
IMpower131	NCT02367794	334	334	23	5	4	3
KEYNOTE -355	NCT02819518	562	281	14	0	6	0
KEYNOTE-119	NCT02555657	309	292	6	0	3	0
DANUBE	NCT02516241	345	313	6	1	2	0
KEYNOTE-181	NCT02564263	314	296	15	2	N. R	N. R
CheckMate 017	NCT01642004	131	129	6	0	1	0
CheckMate 057	NCT01673867	287	268	8	1	3	1
CheckMate 078	NCT02613507	337	165	15	0	4	0
CheckMate 026	NCT01927419	267	263	14	1	6	0
CheckMate 066	NCT01721772	206	205	3	0	0	0
CheckMate 037	NCT01721746	268	102	5	0	0	0

Notes: Study ID: from ClinicalTrials.gov.**;** Exp: experimental; Con: control; N.R: not report. No.: the number of patients.

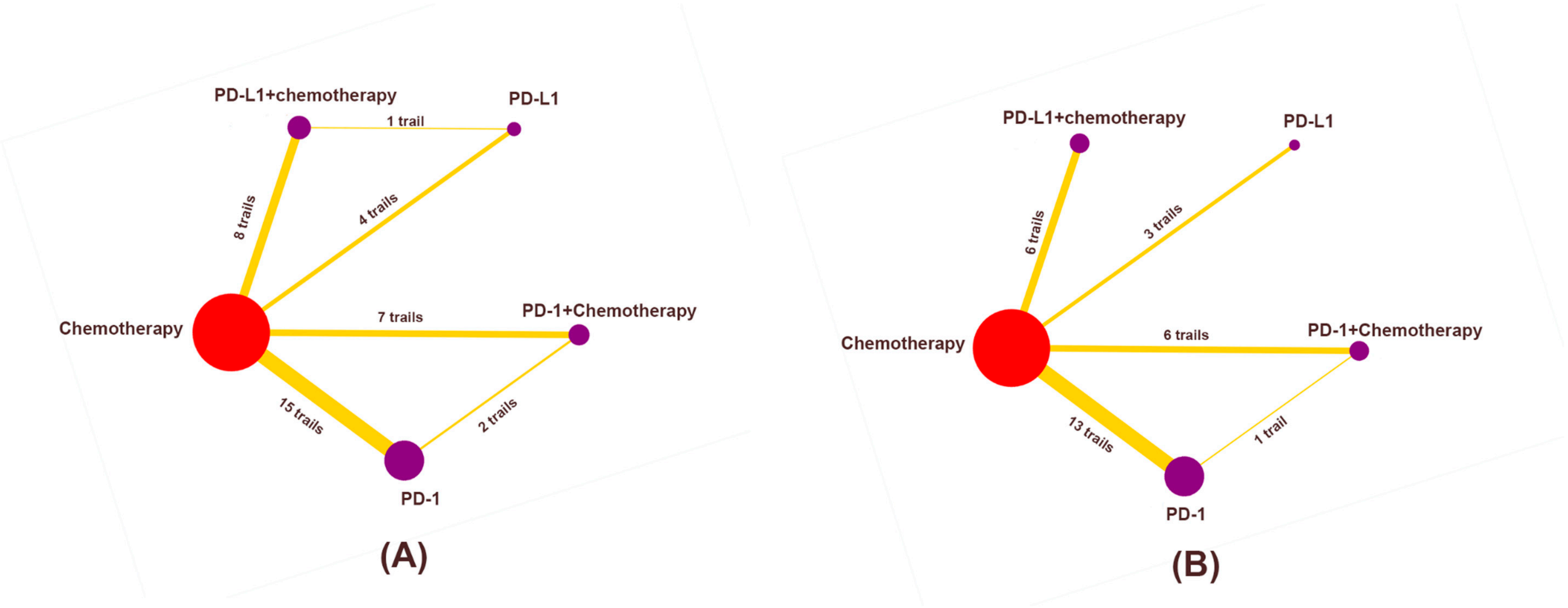

Figure S1. Network plots of subgroup analysis based on different ICIs (A) any grade IRP, (B) grade 3-5 IRP

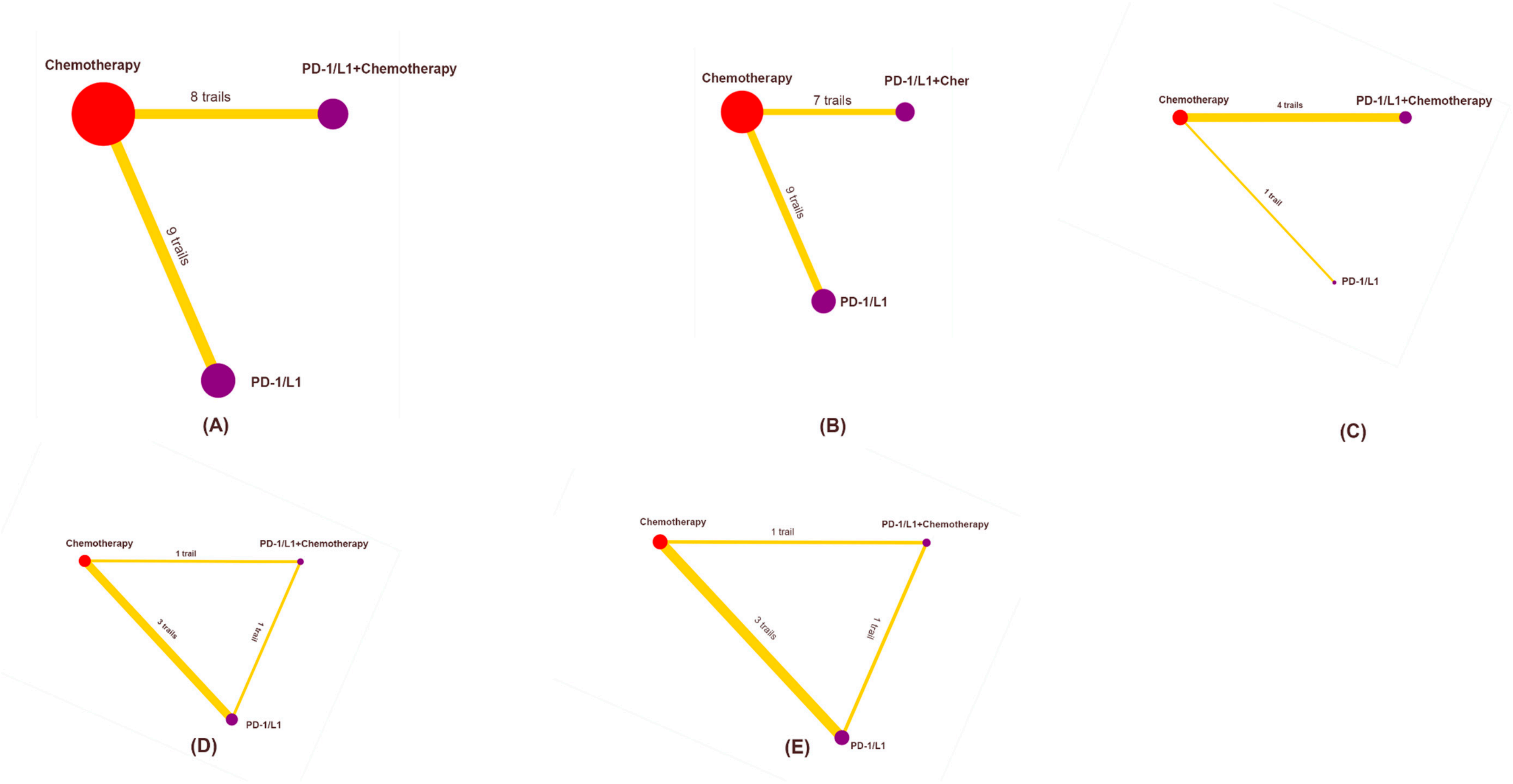

Figure S2. Network established for comparisons based on cancer type. (A) any grade IRP and (B) grade 3-5 IRP in NSCLC, (C) any grade IRP and grade 3-5 IRP in TNBC, (D) any grade IRP in UC, (E) any grade IRP in G/GEJ

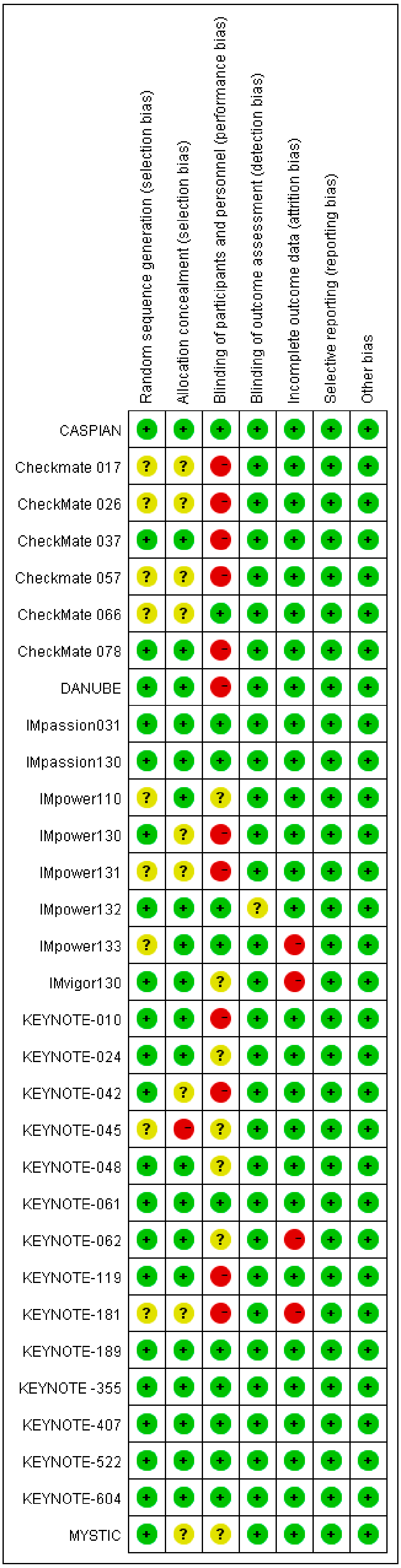

Figure S3. Risk of bias summary: review author's judgements about each risk of bias item for each included study.

Study or Subgroup	ICI monotherapy Events	ICI monotherapy Total	Chemotherapy Events	Chemotherapy Total	Weight	Risk Ratio M-H, Fixed, 95% CI
Checkmate 017	6	131	0	129	1.4%	12.80 [0.73, 224.96]
CheckMate 026	14	267	1	263	2.9%	13.79 [1.83, 104.12]
CheckMate 037	5	268	0	102	2.1%	4.21 [0.23, 75.49]
Checkmate 057	8	287	1	268	3.0%	7.47 [0.94, 59.33]
CheckMate 066	3	206	0	205	1.4%	6.97 [0.36, 134.02]
CheckMate 078	15	337	0	165	1.9%	15.22 [0.92, 252.89]
DANUBE	6	345	1	313	3.0%	5.44 [0.66, 44.97]
IMpower110	11	286	1	263	3.0%	10.12 [1.31, 77.81]
IMvigor130	12	354	6	390	16.4%	2.20 [0.84, 5.81]
KEYNOTE-010	40	682	6	309	23.7%	3.02 [1.29, 7.05]
KEYNOTE-024	12	154	1	150	2.9%	11.69 [1.54, 88.78]
KEYNOTE-042	53	636	3	615	8.8%	17.08 [5.37, 54.38]
KEYNOTE-045	11	267	1	245	3.0%	10.09 [1.31, 77.61]
KEYNOTE-061	8	294	0	276	1.5%	15.96 [0.93, 275.26]
KEYNOTE-062	9	254	1	244	2.9%	8.65 [1.10, 67.73]
KEYNOTE-119	6	309	0	292	1.5%	12.29 [0.70, 217.14]
KEYNOTE-181	15	314	2	296	5.9%	7.07 [1.63, 30.65]
MYSTIC	8	369	5	352	14.7%	1.53 [0.50, 4.62]
**Total (95% CI)**		**5760**		**4877**	**100.0%**	**6.28 [4.36, 9.05]**
Total events	242		29			

Heterogeneity: Chi² = 19.33, df = 17 (P = 0.31); I² = 12%
Test for overall effect: Z = 9.87 (P < 0.00001)

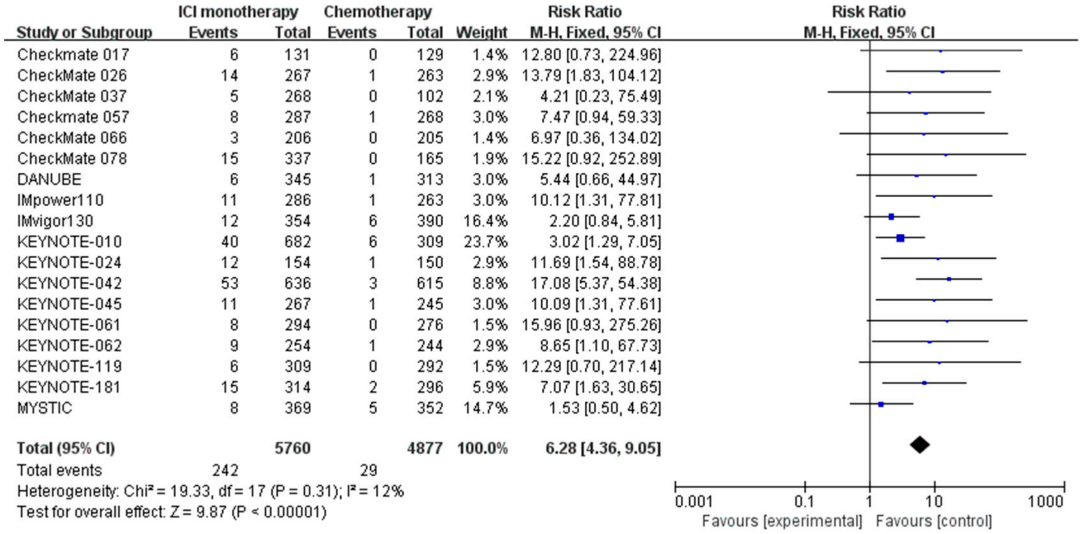

Study or Subgroup	ICIs+Chemotherapy Events	ICIs+Chemotherapy Total	Chemotherapy Events	Chemotherapy Total	Weight	Risk Ratio M-H, Fixed, 95% CI
CASPIAN	7	265	2	266	3.5%	3.51 [0.74, 16.76]
IMpassion031	2	164	2	167	3.5%	1.02 [0.15, 7.14]
IMpassion130	16	453	3	232	7.0%	2.73 [0.80, 9.28]
IMpower130	31	473	5	334	10.4%	4.38 [1.72, 11.14]
IMpower131	23	334	6	274	11.7%	3.14 [1.30, 7.61]
IMpower132	18	291	5	196	10.6%	2.42 [0.92, 6.42]
IMpower133	5	198	1	11	3.4%	0.28 [0.04, 2.18]
IMvigor130	12	453	1	15	3.4%	0.40 [0.06, 2.86]
KEYNOTE -355	14	562	0	281	1.2%	14.53 [0.87, 242.62]
KEYNOTE-189	20	405	6	202	14.2%	1.66 [0.68, 4.08]
KEYNOTE-407	23	278	6	280	10.6%	3.86 [1.60, 9.34]
KEYNOTE-522	10	781	5	389	11.8%	1.00 [0.34, 2.89]
KEYNOTE-604	9	223	5	223	8.8%	1.80 [0.61, 5.29]
**Total (95% CI)**		**4880**		**2870**	**100.0%**	**2.54 [1.86, 3.47]**
Total events	190		47			

Heterogeneity: Chi² = 16.95, df = 12 (P = 0.15); I² = 29%
Test for overall effect: Z = 5.86 (P < 0.00001)

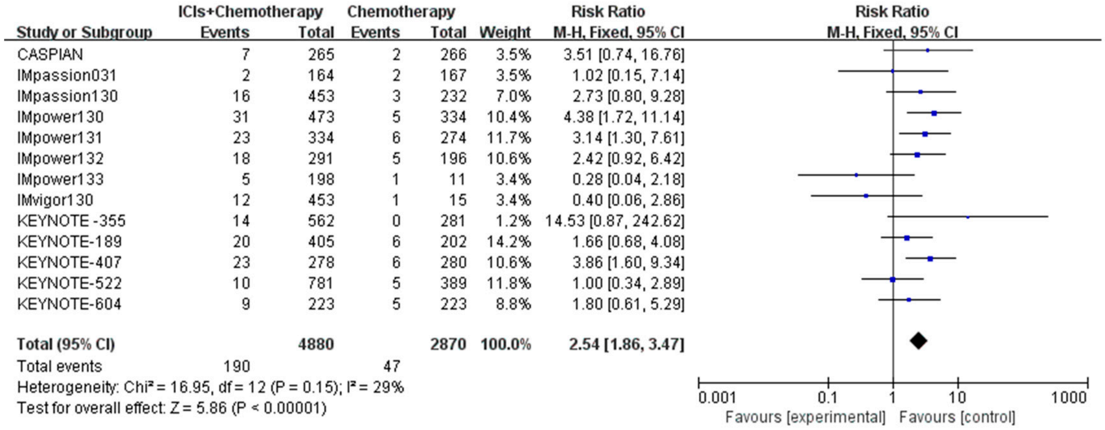

Study or Subgroup	ICIs+Chemotherapy Events	ICIs+Chemotherapy Total	ICIs monotherapy Events	ICIs monotherapy Total	Weight	Risk Ratio M-H, Fixed, 95% CI
IMvigor130	12	453	12	354	48.7%	0.78 [0.36, 1.72]
KEYNOTE-048	2	48	19	300	19.0%	0.66 [0.16, 2.73]
KEYNOTE-062	8	250	9	254	32.3%	0.90 [0.35, 2.30]
**Total (95% CI)**		**751**		**908**	**100.0%**	**0.80 [0.46, 1.39]**
Total events	22		40			

Heterogeneity: Chi² = 0.14, df = 2 (P = 0.93); I² = 0%
Test for overall effect: Z = 0.80 (P = 0.42)

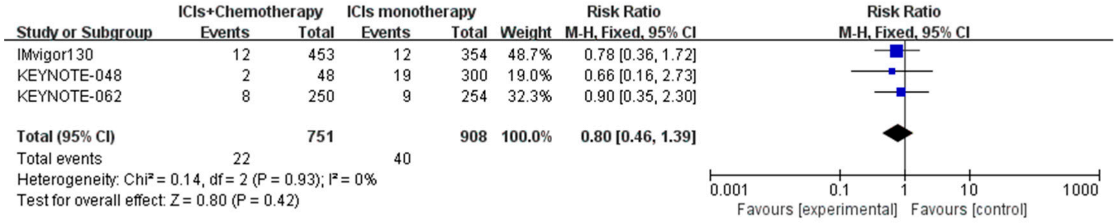

Figure S4. Forest plots and pairwise meta-analysis of direct comparisons for the risk of any grade IRP. ICIs, immune checkpoint inhibitors; IRP, immune-related pneumonitis.

Study or Subgroup	ICIs monotherapy Events	Total	Chemotherapy Events	Total	Weight	Risk Ratio M-H, Fixed, 95% CI
Checkmate 017	1	131	0	129	4.2%	2.95 [0.12, 71.86]
CheckMate 026	6	267	0	263	4.2%	12.81 [0.73, 226.18]
CheckMate 037	0	268	0	102		Not estimable
Checkmate 057	3	287	1	268	8.5%	2.80 [0.29, 26.77]
CheckMate 066	0	206	0	205		Not estimable
CheckMate 078	4	337	0	165	5.5%	4.42 [0.24, 81.61]
DANUBE	2	345	0	313	4.3%	4.54 [0.22, 94.15]
IMpower110	2	286	0	263	4.3%	4.60 [0.22, 95.36]
KEYNOTE-010	18	682	2	309	22.7%	4.08 [0.95, 17.47]
KEYNOTE-024	4	154	1	150	8.3%	3.90 [0.44, 34.46]
KEYNOTE-042	22	636	1	615	8.4%	21.27 [2.88, 157.34]
KEYNOTE-045	6	267	0	245	4.3%	11.93 [0.68, 210.72]
KEYNOTE-061	2	294	0	276	4.2%	4.69 [0.23, 97.36]
KEYNOTE-119	3	309	0	292	4.2%	6.62 [0.34, 127.54]
MYSTIC	5	369	2	352	16.9%	2.38 [0.47, 12.21]
**Total (95% CI)**		**4838**		**3947**	**100.0%**	**5.96 [3.19, 11.12]**
Total events	78		7			

Heterogeneity: Chi² = 4.41, df = 12 (P = 0.97); I² = 0%
Test for overall effect: Z = 5.60 (P < 0.00001)

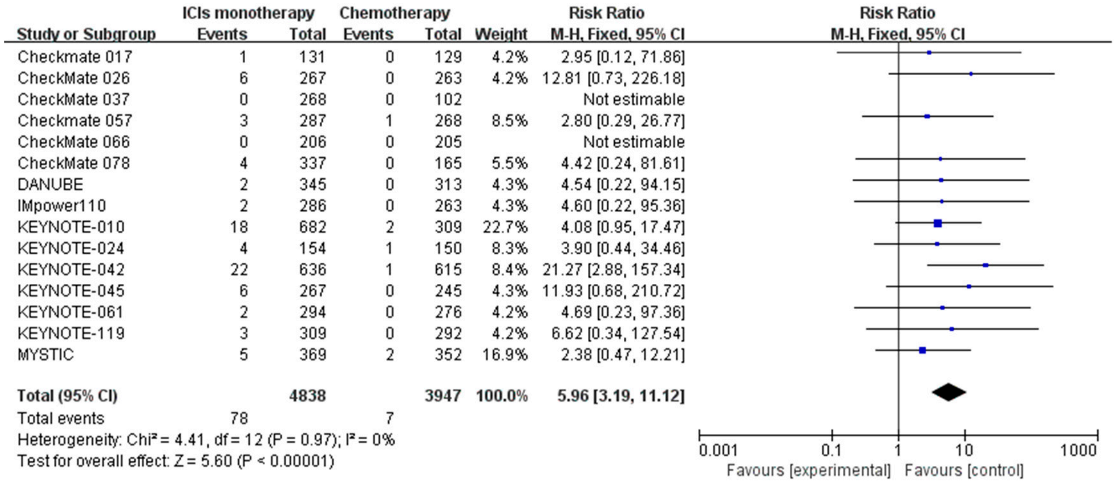

Study or Subgroup	ICIs+Chemotherapy Events	Total	Chemotherapy Events	Total	Weight	Risk Ratio M-H, Fixed, 95% CI
CASPIAN	2	265	1	266	4.8%	2.01 [0.18, 22.01]
IMpassion031	1	164	0	167	2.4%	3.05 [0.13, 74.44]
IMpassion130	2	453	0	437	2.4%	4.82 [0.23, 100.19]
IMpower130	0	473	1	232	9.6%	0.16 [0.01, 4.01]
IMpower131	4	334	3	334	14.3%	1.33 [0.30, 5.91]
IMpower132	6	291	3	274	14.8%	1.88 [0.48, 7.46]
KEYNOTE -355	6	562	0	281	3.2%	6.51 [0.37, 115.18]
KEYNOTE-189	12	405	4	202	25.5%	1.50 [0.49, 4.58]
KEYNOTE-407	9	278	3	280	14.3%	3.02 [0.83, 11.04]
KEYNOTE-522	3	781	1	389	6.4%	1.49 [0.16, 14.32]
KEYNOTE-604	3	223	0	223	2.4%	7.00 [0.36, 134.73]
**Total (95% CI)**		**4229**		**3085**	**100.0%**	**2.05 [1.21, 3.48]**
Total events	48		16			

Heterogeneity: Chi² = 5.11, df = 10 (P = 0.88); I² = 0%
Test for overall effect: Z = 2.68 (P = 0.007)

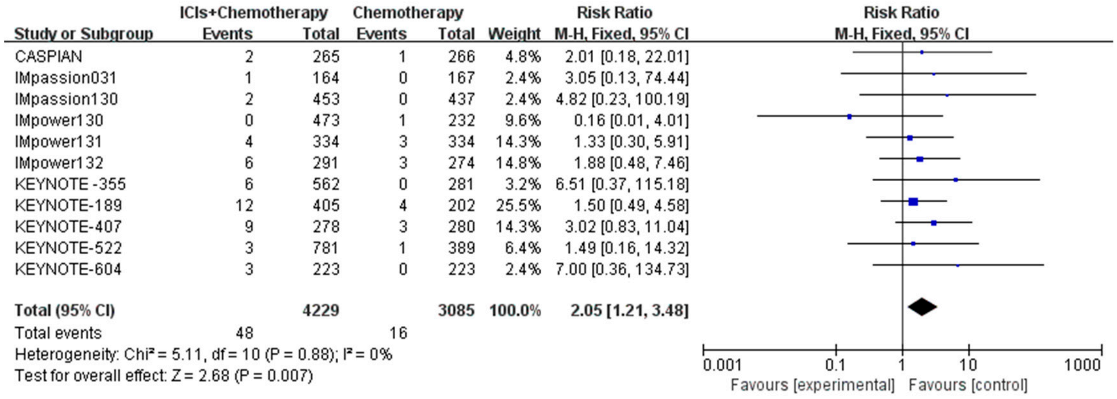

Figure S5. Forest plots and pairwise meta-analysis of direct comparisons for the risk of grade 3 or higher IRP. ICIs, immune checkpoint inhibitors; IRP, immune-related pneumonitis.

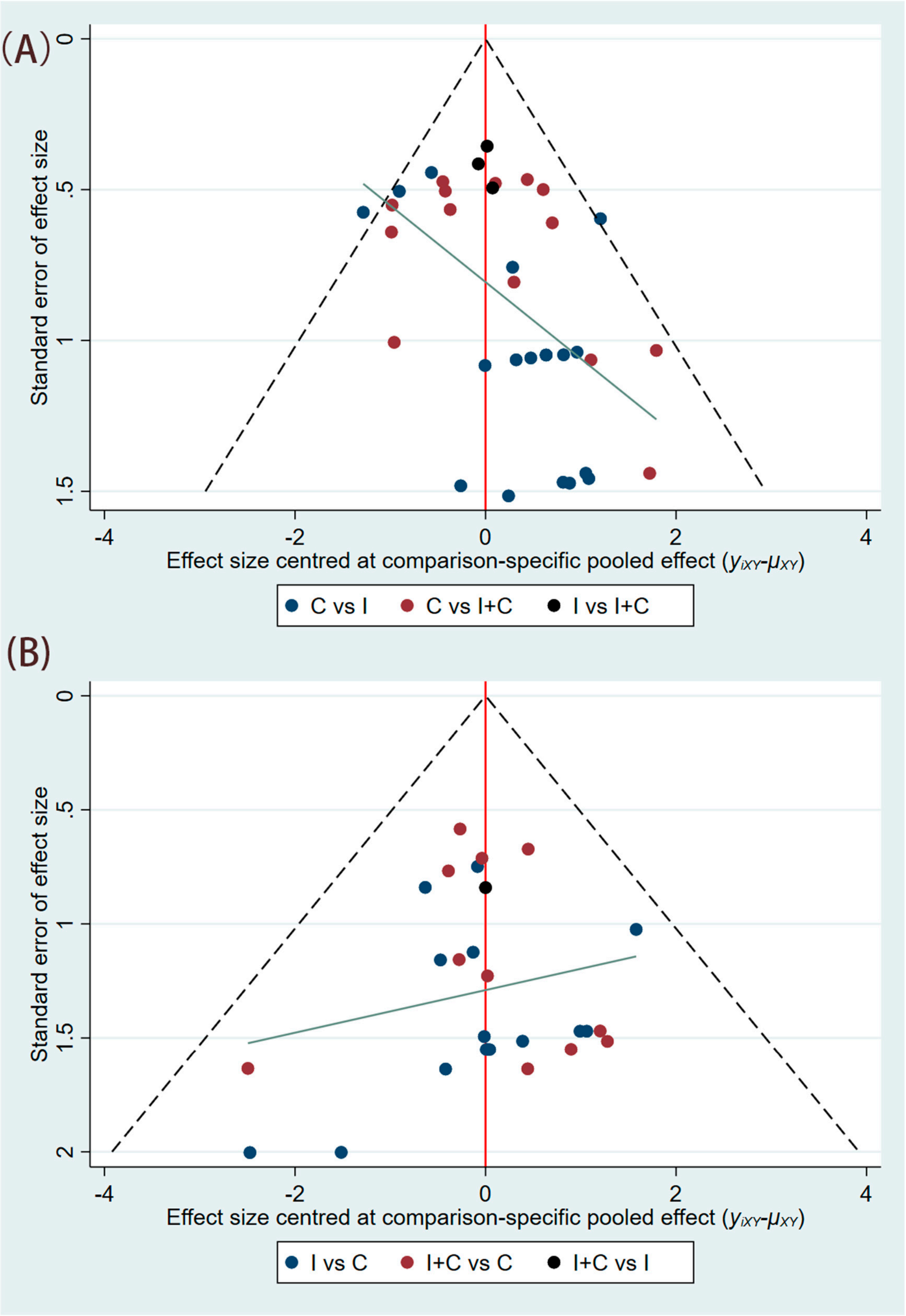

Figure S6. Publication bias for (A) any grade IRP and (B) grade 3-5 IRP in all population.
